# TCF3 Regulates the Proliferation and Apoptosis of Human Spermatogonial Stem Cells by Targeting PODXL

**DOI:** 10.3389/fcell.2021.695545

**Published:** 2021-08-06

**Authors:** Dai Zhou, Jingyu Fan, Zhizhong Liu, Ruiling Tang, Xingming Wang, Hao Bo, Fang Zhu, Xueheng Zhao, Zenghui Huang, Liu Xing, Ke Tao, Han Zhang, Hongchuan Nie, Huan Zhang, Wenbing Zhu, Zuping He, Liqing Fan

**Affiliations:** ^1^Institute of Reproduction and Stem Cell Engineering, School of Basic Medicine Science, Central South University, Changsha, China; ^2^Reproductive & Genetic Hospital of CITIC-Xiangya, Changsha, China; ^3^College of Life Sciences, Hunan Normal University, Changsha, China; ^4^Clinical Research Center for Reproduction and Genetics in Hunan Province, Changsha, China; ^5^NHC Key Laboratory of Human Stem Cell and Reproductive Engineering, Central South University, Changsha, China; ^6^Department of Chemistry and Biochemistry, University of South Carolina, Columbia, SC, United States; ^7^Department of Urology, Hunan Cancer Hospital, Changsha, China; ^8^The Key Laboratory of Model Animals and Stem Cell Biology in Hunan Province, Hunan Normal University School of Medicine, Changsha, China

**Keywords:** TCF3, human spermatogonial stem cells, proliferation, apoptosis, PODXL

## Abstract

Spermatogonial stem cells (SSCs) are the initial cells for the spermatogenesis. Although much progress has been made on uncovering a number of modulators for the SSC fate decisions in rodents, the genes mediating human SSCs remain largely unclear. Here we report, for the first time, that TCF3, a member of the basic helix-loop-helix family of transcriptional modulator proteins, can stimulate proliferation and suppress the apoptosis of human SSCs through targeting podocalyxin-like protein (PODXL). TCF3 was expressed primarily in GFRA1-positive spermatogonia, and EGF (epidermal growth factor) elevated TCF3 expression level. Notably, TCF3 enhanced the growth and DNA synthesis of human SSCs, whereas it repressed the apoptosis of human SSCs. RNA sequencing and chromatin immunoprecipitation (ChIP) assays revealed that TCF3 protein regulated the transcription of several genes, including *WNT2B*, *TGFB3*, *CCN4*, *MEGF6*, and *PODXL*, while PODXL silencing compromised the stem cell activity of SSCs. Moreover, the level of TCF3 protein was remarkably lower in patients with spermatogenesis failure when compared to individuals with obstructive azoospermia with normal spermatogenesis. Collectively, these results implicate that TCF3 modulates human SSC proliferation and apoptosis through PODXL. This study is of great significance since it would provide a novel molecular mechanism underlying the fate determinations of human SSCs and it could offer new targets for gene therapy of male infertility.

## Introduction

Spermatogonial stem cells (SSCs) reside along the basement membrane of the testicular seminiferous tubules, and they self-renew to maintain the stem cell pool of the testis and differentiate for the continuous production of spermatozoa (Kanatsushinohara et al., 2003; Yeh et al., 2011). In both rodents and primates, SSCs are restricted to A-type undifferentiated spermatogonia (A_undiff_). In rodents, A_undiff_ are found as single (A_s_) or as syncytia of 2 (A-paired, A_pr_), 4, 8, and up to 16 (A-aligned, A_al__4__–__16_) cells interconnected by cytoplasmic bridges (Mäkelä and Toppari, 2018). SSCs are generally considered to be present in A_s_ spermatogonia (Lord and Oatley, 2017), however, many data indicate that A_pr_ and A_al_ syncytium can re-enter A_s_ state by breaking the cytoplasmic bridge in an appropriate environment (Hara et al., 2014; Carrieri et al., 2017). In primates, A_undiff_ can be divided into Adark (A_d_) and Apale (A_p_) according to the degree of nuclei staining. The traditional models propose that A_d_ spermatogonia are the reserve stem cells, and they steadily generate A_p_ spermatogonia with adequate self-renewal potential to sustain the stem cell pool (Clermont, 1966), but there are still many controversies about the function of A_d_ and A_p_ (Hermann et al., 2009, 2010; Di Persio et al., 2017).

Using testis material from rodents, molecular mechanisms of mouse SSC self-renewal and differentiation have been analyzed. It is now considered that the intricate molecular and cellular interactions form the niche or microenvironment for SSC development (He et al., 2008; Makela and Hobbs, 2019). GFRA1-positive SSCs respond to GDNF and other factors through elevating expression of genes required to sustain the self-renewing state of mouse SSCs, including Etv5 (Oatley et al., 2006), Lhx1 (Oatley et al., 2006), Bcl6b (Oatley et al., 2006), Nanos2 (Sada et al., 2012), and Id4 (Oatley et al., 2011). The secretion of GDNF is under endocrine modulation in FSH by Sertoli cells as well as PMCs (peritubular myoid cells) through the LH-mediated synthesis of testosterone in Leydig cells (Sakai et al., 2018). FGF2 and GDNF synergistically function to promote the robust growth of the undifferentiated spermatogonia *in vitro*, while the role of FGF2 in sustaining the stemness of SSCs *in vivo* remain poorly understood (Takashima et al., 2015). Shisa6, a WNT suppressor, renders SSCs resistant to differentiation induced by WNT. The progenitor spermatogonia primed for the differentiation are originated from the GFRA1-positive SSCs in response to WNT induction (Tokue et al., 2017). They have adopted the *Ngn3* and *Sox3* gene signatures, which separates them clearly from the self-renewing cells and enables them to be responsive to retinoic acid (RA) through expressing RARγ (retinoic acid receptor gamma) (Sugimoto et al., 2012; Hara et al., 2014; Ikami et al., 2015). The RA induction elicits a differentiation commitment in these cells, resulting in the up-regulation of Stra8, Sohlh1 and Kit as well as the down-regulation of Plzf (Ghyselinck et al., 2006; Anderson et al., 2008; Zhou et al., 2008). Besides, RA acts on Sertoli cells, which enhances the expression level of Bmp4 and decreases the transcription of *Gdnf* (Pellegrini et al., 2008; Carlomagno et al., 2010; Yang et al., 2013).

Although much progress has been achieved on unveiling the molecular mechanisms underlying the self-renewal and the differentiation of SSCs in rodents, these rodent SSC mechanisms cannot be repeated in human SSCs. The cell types, as well as the biochemical phenotypes of SSCs are distinct in SSCs between rodents and humans. It is worth noting that human SSCs share some but not all phenotypes with rodent SSCs. As an example, OCT4 (also known as POU5F1) is a hallmark for rodent SSCs; however, OCT4 is not expressed in human SSCs (He et al., 2010; Guo et al., 2017). Moreover, the mouse seminiferous epithelium cycle is usually subdivided into 12 stages, while there are generally only 6 spermatogenic cycle phases in human (Muciaccia et al., 2013). Consequently, the regulatory mechanisms of SSCs have significant differences between humans and rodents. As such, it is of great significance for us to identify the novel molecular regulators modulating the fate determinations of human SSCs.

TCF3 belongs to the E-protein family, the highly conserved bHLH (basic helix-loop-helix) family members of transcriptional modulator proteins (Slattery et al., 2008). TCF3 participates in diverse developmental processes through working with other HLH family proteins (Imayoshi and Kageyama, 2014). It has been reported that TCF3 plays pivotal roles in the stem cell maintenance and differentiation, including the embryonic stem cells, neural stem cells, and hematopoietic stem cells. The alternative splicing of TCF3 regulated by hnRNP H/F modulates the expression of E-cadherin and pluripotency of human embryonic stem cells (Yamazaki et al., 2018). TCF3 maintains the hematopoietic stem cell arsenal and enhances the maturation of myeloerythroid and myelo lymphoid progenitors (Semerad et al., 2009), while TCF3 controls the differentiation of neural stem cells into astrocytes (Bohrer et al., 2015). Moreover, TCF3 proteins trigger and suppress transcription so as to enhance a B cell fate in progenitor cells (Lin et al., 2010). These studies imply that TCF3 is associated with cell fate decisions on various kinds of stem cells.

In the current study, we revealed that TCF3 was mainly expressed in the human SSCs (GFRA1^+^/PCNA^+^/KIT^–^) by using immunohistochemistry. We used RNA interference to explore the function of TCF3 in human SSCs, and we demonstrated that TCF3 promoted the proliferation and DNA synthesis but inhibited the apoptosis of human SSC line. In order to identify the targets ofTCF3, RNA sequencing and chromatin immunoprecipitation (ChIP) approaches were employed. TCF3 could directly docks to the promoter region and enhanced the expression levels of podocalyxin-like protein (PODXL), TGFB3, CCN4, and MEGF6. Defective PODXL expression inhibited the proliferation and DNA synthesis but promoted apoptosis of human SSC line. In addition, we revealed that TCF3 levels were remarkably decreased in NOA patients compared to individuals with obstructive azoospermia (OA) with normal spermatogenesis, particularly in the NOA patients with spermatogonial or spermatocytes maturation arrest (Spc MA). Therefore, our study provides novel insights into the molecular mechanisms responsible for the proliferation and the apoptosis of human SSCs, and it provides new clues for the etiology, molecular diagnosis, as well as the treatment of male infertility.

## Materials and Methods

### Human Testis Tissues

This study was approved by the Ethics Committee of the Reproductive & Genetic Hospital of CITIC-Xiangya, Basic Medical Science School, Central South University (LL-SC-2017-015), and the participants signed an informed consent. Adult testicular tissues retrieved for this research were obtained from 12 patients (3 OA and 9 NOA) undergoing conventional testicular sperm extraction (TESE) or microdissection testicular sperm extraction (m-TESE) aged between 25 and 38 years old, and about 50 mg testicular tissues were acquired. Normal fetal testes for this research were obtained from two donors (19 and 24 weeks). All the samples were washed three times by PBS with 1% penicillin and streptomycin and then fixed with 4% paraformaldehyde (PFA) or stored in liquid nitrogen.

### Human SSC Line Culture

The human SSC line was established by overexpressing human SV40 large T antigen in human primary GPR125-positive undifferentiated spermatogonia in our lab (Hou et al., 2015), the cell line was positive for a series of SSC markers including GPR125, GFRA1, PLZF, UCHL1, and THY1, and it can be expanded *in vitro* for a long time.

The human SSC line cells were cultured in DMEM/F12 (Gibco, Grand Island, NY, United States) with 10% FBS (Gibco) and 100 unit/mL streptomycin/penicillin (Invitrogen, CA, United States) at 34°C in 5% CO_2_ incubator. The cells were passaged every 4 days using 0.05% trypsin and 0.53 mM EDTA (Invitrogen). In order to seek which growth factor mediates TCF3, the cells were incubated in the DMEM/F12 with the addition of 10 ng/mL GDNF (R&D Systems, MN, United States), 10 ng/mL FGF2 (R&D Systems), 10 ng/mL epidermal growth factor (EGF) (Sigma, MO, United States), or 1,000 IU/mL LIF (Cyagen, Suzhou, China).

### RNA Isolation, RT-PCR, and qPCR

The RNAiso Plus reagent (Takara, Kusatsu, Japan) was employed to isolate total RNA from the cells pursuant to the instruction of the manufacturer. The quality and concentrations of total RNA were determined using the Nanodrop (Thermo Scientific, MA, United States), and cDNA was generated from total RNA by using Transcriptor First Strand cDNA Synthesis Kit (Roche, Mannheim, Germany).

Real-time PCR (RT-PCR) was performed in terms of the method as documented previously (Zhou et al., 2020). The PCR reactions were conducted as follows: 95°C for 5 min, denaturation at 95°C for 30 s, annealing at 52–60°C for 45 s as indicated in Supplementary Table 1, and elongation at 72°C for 45 s for 32 cycles. RNA without room temperature (RT) was employed as the negative control (NC). Electrophoresis of the PCR amplicons on a 2% agarose gel was performed and ethidium bromide was employed to stain the amplicons. The chemiluminescence imaging system (Chemi-Doc XRS, Bio-Rad, CA, United States) was employed to acquire the images of the bands.

The SYBR Premix Ex Taq II (Takara) was employed to perform qPCR on the Applied Biosystems ABI Prism 7700 system (Applied Biosystems, Foster City, CA, United States) pursuant to the manual of the manufacturer. *ACTB* served as the normalization standard. The 2^–ΔΔ^ cycle threshold (Ct) approach was utilized to determine relative mRNA expression. Each sample was replicated three times. Primer sequences of the chosen genes for RT-PCR and qPCR were designed and shown in Supplementary Table 1.

### Immunocytochemistry, Immunohistochemistry, and Immunofluorescence

For the immunocytochemistry, the cells were washed three times with cold PBS (Gibco), and they were fixed with 4% PFA for 15 min. After washing three times in cold PBS, 0.25% Triton X-100 (Sigma) was employed to permeabilize the cells for 10 min. Blocking was performed by 5% BSA at RT for 1 h, and the cells were incubated with the primary antibodies at 4°C overnight. The sources and dilutions of the antibodies were shown in Supplementary Table 2. Next, the cells were rinsed with PBS and then incubated with Alexa Fluor 488 labeled IgG or Alexa Fluor 594 labeled IgG secondary antibodies. Thereafter, DAPI staining was used to stain the nucleus of the cells. A fluorescence microscope (Carl Zeiss, Oberkochen, Germany) was employed to capture image of the cells.

For the immunohistochemistry, deparaffinization of the testis sections was conducted with xylene and rehydrated with the graded ethanol. Heat-induced antigen retrieval (HIER) was done in 0.01M sodium citrate buffer in a beaker at 98°C for 18 min, and 3% H_2_O_2_ (Zsbio, Beijing, China) was utilized to block the endogenous peroxidase activity. Thereafter, 0.25% Triton X-100 (Sigma) was used to permeabilize the cell sections for 15 min, and 5% BSA was employed to block these sections for 1 h at RT. Subsequently, the sections were incubated with primary antibodies at 4°C overnight and followed by rinsing in PBS. Next, the sections were incubated with HRP-labeled secondary antibody for 1 h at RT, and chromogen was detected with the 3,3′-diaminobenzidine (DAB) chromogen kit (Dako, Glostrup, Denmark). The sections were finally stained with hematoxylin. For immunofluorescence, the sections were incubated with Alexa Fluor conjugated second antibody for 1 h at RT, and the cell nuclei were stained with DAPI. The images were acquired using a Zeiss microscope.

### Western Blots

Testis tissues and human SSC line were lysed with the RIPA lysis buffer (Thermo Scientific) on ice for about 30 min, and cell lysates were removed by spinning at 12,000 × *g*. The BCA kit (Thermo Scientific) was employed to determine concentration of the proteins. For every sample, 30 μg of total protein extracts were fractionated on the SDS-PAGE Gel (Bio-Rad), and western blotting was performed in terms of the method as documented previously (Zhou et al., 2020). The antibodies and the dilution ratios for the western blotting were shown in Supplementary Table 2. The chemiluminescence (Bio-Rad) was employed to visualize the protein bands.

### siRNA Transfection

The siRNA sequences targeting human TCF3 and PODXL mRNA were synthesized by Ribobio (Guangzhou, China) and were listed in Supplementary Table 3. The siRNA without targeting sequences were used as NCs. Cells were transfected with 100 nM siRNA or NC-siRNA using the Lipofectamine 3000 transfection system (Cat No, Life Technologies, CA, United States) according to the manual of the manufacturer. Cells were harvested after transfection for 48 h to assess the alterations of gene and protein expression.

### CCK-8 Assay

After transfection with siRNAs, human SSC proliferation potential was explored with the CCK-8 assay Kit (Dojindo, Kumamoto, Japan) as described by the manufacturer. Concisely, cell growth medium was replaced with 10% CCK-8 reagents and incubated for 3 h, and the OD values were read at 450 nm on a microplate reader (Thermo Scientific).

### EdU Incorporation Assay

In total, 5,000 human SSC line/well were seeded to 96-well plate in DMEM/F12 medium with addition of 50 μm EdU (RiboBio) for 12 h. The cells were rinsed with DMEM and fixed with 4% PFA. Thereafter, 2 mg/mL glycine was employed to neutralize the cells and then permeabilization was performed for 10 min using 0.5% Triton X-100 at RT. Apollo staining reaction buffer was employed for EdU immunostaining, and Hoechst 33342 was used to stain the cell nuclei. The images were captured using a fluorescence microscope (Zeiss), and at least 500 cells were counted to assess the percentages of EdU-positive cells.

### Flow Cytometry With Annexin V-APC/PI Staining

To assess the apoptosis of the human SSC line affected by TCF3 siRNAs, cells were digested and rinsed twice in ice-cold PBS. Afterward, 10^6^ cells were re-suspended in Annexin V Binding Buffer (BD Biosciences, NJ, United States) in terms of the methods of the manufacturer. The cells were incubated with 5 μl of Annexin V labeled APC and 10 μl of PI solution for 15 min at RT in dark. A C6 flow cytometry instrument (BD Biosciences) was employed to analyze the cells.

### TUNEL Assay

The *In Situ* Cell Death Detection Kit (Roche) was further employed to assess the apoptosis of the human SSC line affected by TCF3 siRNAs pursuant to the manual of the manufacturer. The cells were treated with 20 mg/mL proteinase K for 15 min at RT, and they were incubated with dUTP labeling/terminal deoxynucleotidyl transferase (TdT) enzyme buffer for 1 h in the dark. DAPI was used to stain the cell nuclei. The cells with PBS but without the TdT enzyme was employed as the NC. At least 500 cells were evaluated per sample in a Zeiss fluorescence microscope.

### RNA Sequencing

The Trizol reagent kit (Invitrogen) was employed to isolate total RNA, and the Agilent 2100 Bioanalyzer (Agilent Technologies, CA, United States) was used to measure RNA quality and checked by RNase free agarose gel electrophoresis. The Oligo (dT) beads were utilized to enrich the eukaryotic mRNA, while the Ribo-ZeroTM Magnetic Kit (Epicentre, WI, United States) was employed to remove rRNA to enrich prokaryotic mRNA. Next, the fragmentation of the enriched mRNA into short fragments was conducted with the fragmentation buffer and followed by reverse transcription into cDNA using random hexamers. DNA polymerase I, dNTP, buffer, and RNase H were employed to synthesize the second strand DNA. Subsequently, the QiaQuick PCR extraction kit (Qiagen, Venlo, Netherlands) was sued to purify the cDNA fragments, followed by their end repair, poly(A) introduction, and ligation with Illumina sequencing adapters. Thereafter, agarose gel electrophoresis was performed to select the ligation products, which were enriched *via* PCR, and sequenced on the Illumina HiSeq2500 system by Gene *Denovo* Biotechnology Co. (Guangzhou, China). Reads obtained from the sequencing machine were filtered by fastp (Chen et al., 2018) (version 0.18.0). The rRNA mapped reads were removed by using a short reads alignment tool Bowtie2 (Langmead and Salzberg, 2012) (version 2.2.8). The remaining clean reads were further employed in assembly and the gene abundance determination. The THISAT (Kim et al., 2015) with “-rna-strandness RF” and other parameters set as a default was employed to map the paired-end clean reads to the reference genome. The StringTie v1.3.1 (Pertea et al., 2015, 2016) was used to assemble the mapped reads in a reference-based strategy. RNA differential expression assessment was conducted by DESeq2 (Love et al., 2014) software, and FDR <0.05 along with absolute fold change ≥2 indicated the differentially expressed genes (DEGs) (Supplementary Data Sheet 1). The Gene Ontology (GO) (Ashburner et al., 2000) and Kyoto Encyclopedia of Genes and Genomes (KEGG) pathway enrichment analyses (Kanehisa and Goto, 2000) were implemented on the DEGs in human SSC line between TCF3-siRNA 3 and the NC-siRNA.

### ChIP Assay and Real-Time PCR

The SimpleChIP^®^ Plus Sonication Chromatin IP Kit (Cell Signaling Technology, MA, United States) was employed to perform ChIP assay. In brief, about 1 × 10^7^ cells were fixed with 1% formaldehyde, then ice-cold 1.25 M glycine was used to quench the cells, which were re-suspended and lysed. The pellets of cells were re-suspended in 400 μl of shearing buffer enriched with Protease/Phosphatase Inhibitor Cocktail (Cell Signaling Technology). Thereafter, sonication was done to shear cross-linked DNA to an optimal fragment of 200–1,000 bp. The sonicated chromatins were confirmed by assessing size distribution on a 1.5% agarose gel. Immunoprecipitation, de-cross linking, and DNA purification steps were performed in terms of the instruction of the manufacturer. In total, 10 μg of sheared chromatin was incubated with TCF3 antibody (2 μg, Santacruz, sc-133075) and normal mouse IgG (2 μg, Cell Signaling Technology, 5873). After being incubated with antibodies at 4°C overnight, ChIP-Grade Protein G Magnetic Beads was added for 2 h. The Protein G Magnetic Beads were filtered through placing the tubes in a magnetic separation rack for 2 min for the solution to clear. The cross-links were reversed by addition of 6 μl 5 M NaCl and 2 μl Proteinase K and incubated for 2 h at 65°C. The DNA purification spin columns (Cell Signaling Technology) were employed to purify the DNA. The enrichment of particular DNA sequences during immunoprecipitation was subjected to RT-PCR with ChIP PCR primers. The sequences of the primers are shown in Supplementary Table 4.

### Statistical Analysis

Descriptive and statistical analyses were implemented in the GraphPad Prism 8.0 (GraphPad software, CA, United States). Each experiment was replicated three times, and all values are presented as the mean ± SD. The *t*-test was employed to calculate the statistical difference between two groups, and the *p* < 0.05 was considered the statistical significance.

## Results

### TCF3 Is Primarily Expressed in Human Spermatogonial Stem Cells

**FIGURE 1:**
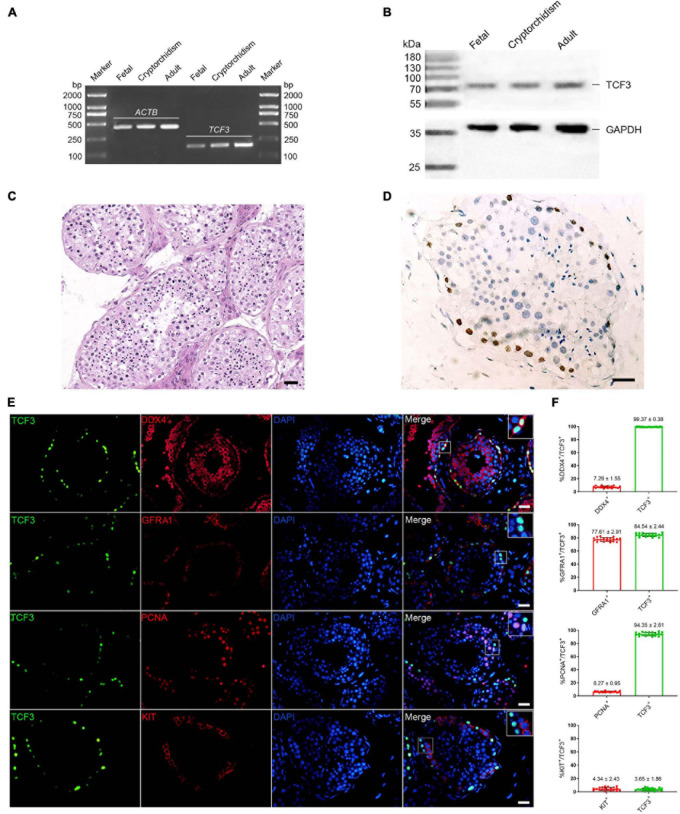
The expression of TCF3 in human testes. **(A)** RT-PCR showed *TCF3* mRNA in human fetal, cryptorchidism, and adult testes. *ACTB* served as the loading control of total RNA. **(B)** Western blotting illustrated the expression of TCF3 protein in human fetal, cryptorchidism, and adult testes. GAPDH was used as the loading control of total protein. **(C,D)** Immunohistochemistry revealed cellular localization of TCF3 in human testis with normal spermatogenesis. **(E)** Double immunostaining showing the co-expression of TCF3 with DDX4, GFRA1, KIT, and PCNA in human testis with normal spermatogenesis. At least 20 seminiferous tubules were counted. **(F)** Percentages of TCF3^+^ cells with DDX4, GFRA1, PCNA, and KIT expression. Scale bars: **(C–E)**, 50 μm.

We first examined TCF3 expression in human fetal and adult testes. RT-PCR and western blots revealed that *TCF3* transcript (Figure 1A) and TCF3 protein (Figure 1B) were detected in both fetal and adult testes. In fetal testes, most of the gonocytes (precursors of spermatogonia) expressed TCF3. TCF3 expression was more frequently found in DDX4-positive cells than KIT-positive cells, and only 24.19% of TCF3-expressing cells have proliferation ability, as evidenced by the PCNA^+^ cells (Supplementary Figure 1). In normal adult human testes (Figure 1C), TCF3 was expressed in the nuclei of spermatogonia along the basement membrane of the seminiferous tubules (Figure 1D). Immunofluorescence further showed that 99.37% of TCF3-expressing cells were DDX4-positive germ cells and 84.5% of TCF3-expressing cells were GFRA1-positive spermatogonia. TCF3 was barely found in the KIT-expressing cells. Interestingly, PCNA was found in 94.35% of TCF3-expressing cells (Figures 1E,F), which suggests that TCF3 may be involved in cell proliferation in human adult testes. Together, these data implicate that TCF3 is primarily expressed in human SSCs with proliferation potential.

**FIGURE 2:**
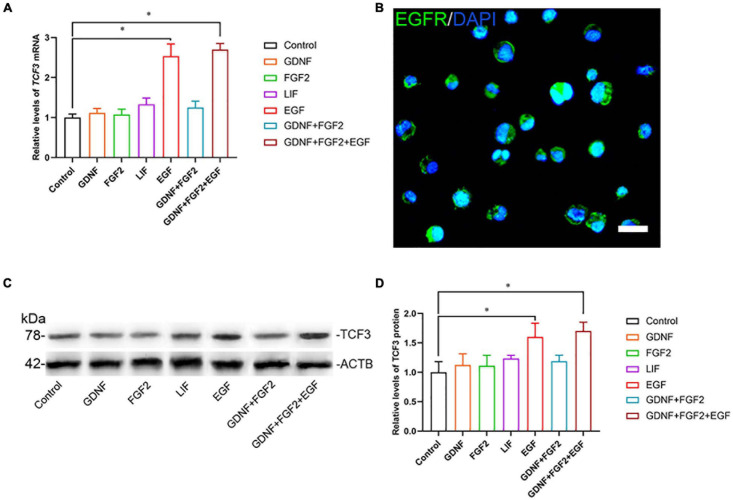
The effect of growth factors GDNF, FGF2, LIF, and EGF on TCF3 expression. **(A)** qPCR assessment of mRNA levels of *TCF3* by growth factors GDNF, FGF2, LIF, and EGF in human SSC line. **(B)** Immunofluorescence showed the presence of EGFR in human SSC line. **(C,D)** Western blotting displayed TCF3 levels by several growth factors. Scale bar: **(B)**, 20 μm. ^∗^*p* < 0.05 indicated the significant differences between the growth factor and the control.

To explore the function and mechanism of TCF3 protein, the human SSC line was utilized. We checked the identity of the human SSC line, and it expressed numerous markers for human primary SSCs, including GFRA1, PLZF, VASA, GPR125, UCHL1, RET, and THY1 (Supplementary Figures 2A–C). Additionally, some hallmarks for human Sertoli cells, including GATA4 and SOX9, were not detected in this cell line (Supplementary Figures 2A–C). To ascertain which growth factors regulate TCF3, GDNF, FGF2, EGF, or LIF was added to the culture medium of human SSC line. RT-PCR (Figure 2A) and western blots (Figures 2C,D) showed that EGF elevated the levels of TCF3 in human SSC line, whereas there was no obvious difference in the levels of TCF3 by GDNF, FGF2, or LIF (Figures 2A,C,D). We also found the expression of EGFR in human SSC line by using immunofluorescence (Figure 2B). These results suggest that TCF3 was modulated by EGF rather than by LIF, FGF2, or GDNF in the human SSC line.

### TCF3 Knockdown Suppresses the Proliferation and Promotes the Apoptosis of Human SSC Line

**FIGURE 3:**
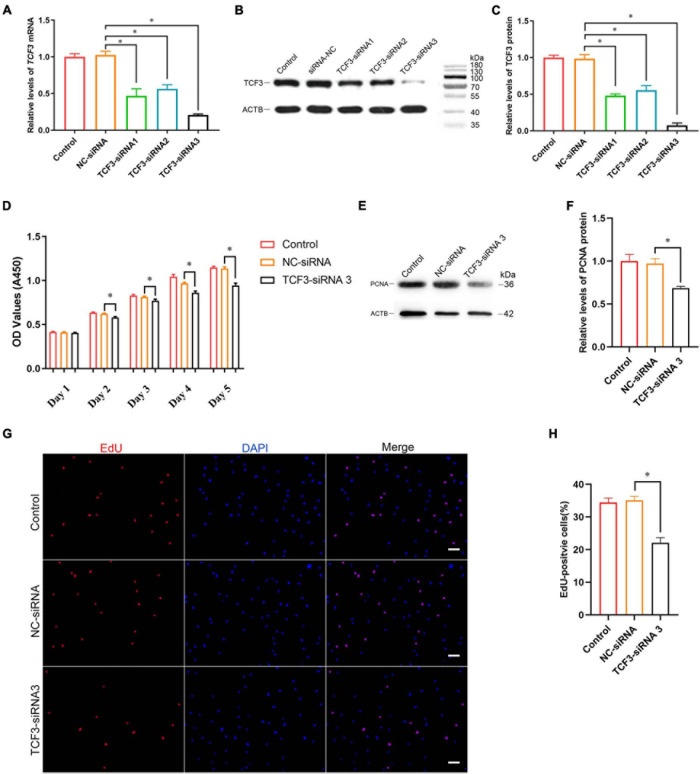
The influence of TCF3 knockdown on human SSC line proliferation. **(A)** qPCR revealed *TCF3* mRNA expression changes in human SSC line by the TCF3-siRNA 1-, 2-, and 3. **(B,C)** Western blotting displayed TCF3 protein expression alterations in human SSC line after the transfection of TCF3-siRNA 1-, 2-, and 3. ACTB was used as the loading control of total protein. **(D)** CCK-8 assay illustrated the proliferation of human SSC line by the (NC-siRNA and TCF3-siRNA 3. **(E,F)** The relative levels of PCNA protein in human SSC line after transfection of NC-siRNA and TCF3-siRNA 3. **(G,H)** The percentages of EdU-positive cells in human SSC line influenced by NC-siRNA and TCF3-siRNA 3. Scale bars: **(G)**, 50 μm. ^∗^*p* < 0.05 denoted the significant differences between TCF3-siRNA 3 and NC-siRNA.

To explore the effect of TCF3 knockdown on SSCs, siRNA-triggered silencing of TCF3 was carried out. RT-PCR and western blotting illustrated that the levels of TCF3 were remarkably reduced by TCF3-siRNA 1, 2, and 3, while TCF3-siRNA 3 had the best silencing effect (Figures 3A–C). The CCK-8 assay displayed that TCF3-siRNA 3 inhibited the proliferation of human SSC line at day 2 to 5 after siRNA transfection (Figure 3D). Western blotting demonstrated that PCNA (cell proliferation hallmark) level was decreased by TCF3-siRNA 3 (Figures 3E,F). Likewise, after transfection for 48 h, the percentages of EdU-positive cells were decreased by TCF3-siRNA 3 compared to the NC-siRNA (35.10 ± 1.21 vs. 22.12 ± 1.54%, *p* < 0.05) (Figures 3G,H). Considered together, these data indicate that TCF3 promotes the proliferation and DNA synthesis of human SSCs.

**FIGURE 4:**
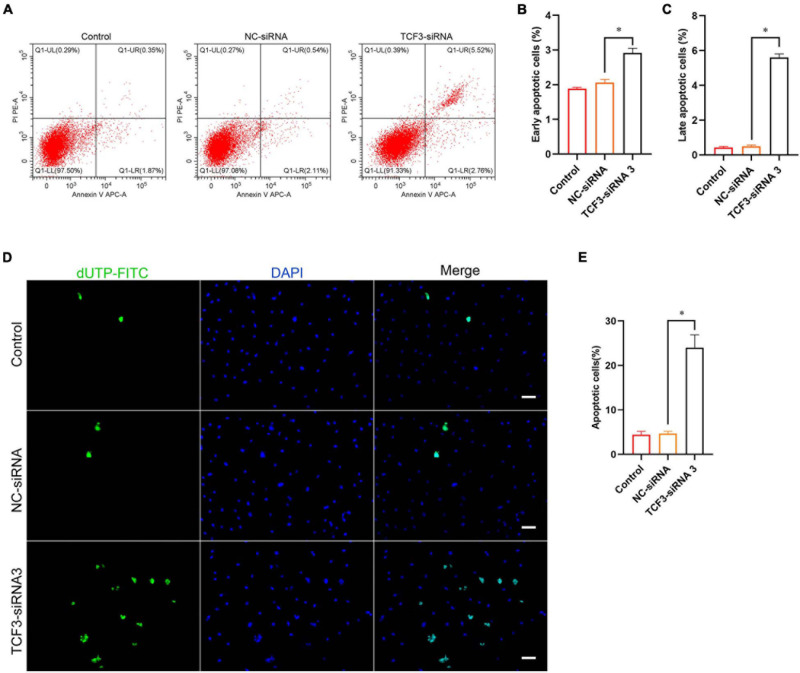
The influence of TCF3 knockdown on the apoptosis of the human SSC cells. **(A–C)** Flow cytometric and APC Annexin V analysis of the percentages of early and late apoptosis in the human SSC line with transfection of NC-siRNA and TCF3-siRNA 3. **(D,E)** TUNEL assays of the percentages of TUNEL^+^ cells in the human SSC line with transfection of NC-siRNA and TCF3-siRNA 3. Scale bars: **(D)**, 50 μm. ^∗^*p* < 0.05 illustrated the significant differences between TCF3-siRNA 3 and NC-siRNA.

Furthermore, Annexin V/PI staining and flow cytometry was used to elucidate the influence of TCF3 on regulating human SSC apoptosis. The percentages of early and late apoptosis in human SSC line were enhanced by TCF3-siRNA 3 compared with the NC-siRNA (Figures 4A–C). Similarly, TUNEL assay showed that the percentages of TUNEL-positive cells were elevated by TCF3-siRNA 3 than NC-siRNA (Figures 4D,E). Taken together, these results reflect that TCF3 knockdown promotes the apoptosis of human SSC line.

### Screening of TCF3 Target Genes

**FIGURE 5:**
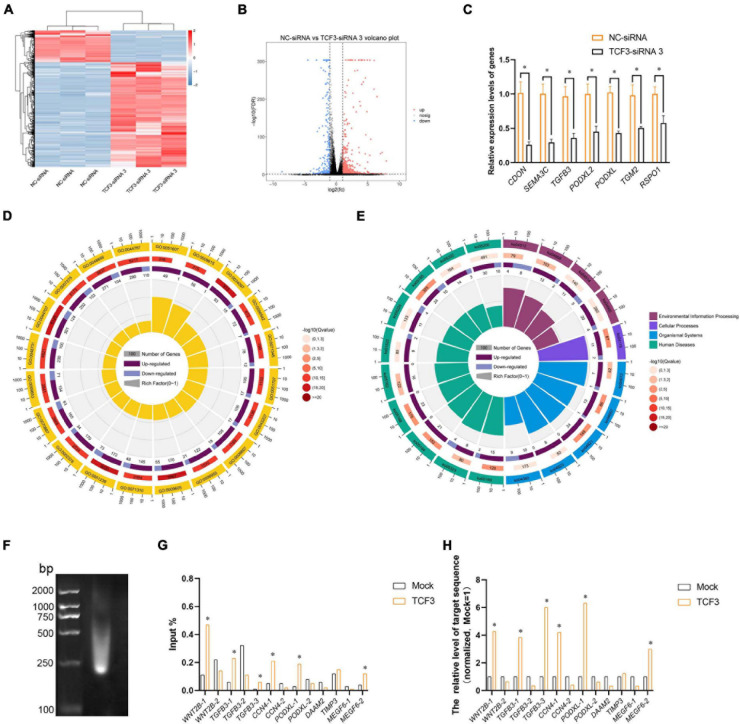
Identification of the target genes of TCF3. **(A)** hierarchical clustering demonstrated the differentially expressed genes (DEGs) in the human SSC line between NC-siRNA and TCF3-siRNA 3. **(B)** Volcano plot illustrated the differentially expressed mRNAs. **(C)** qPCR evaluated the changes in *CDON*, *SEMA3C*, *TGFB3*, *PODXL2*, *PODXL*, *TGM2*, and *RSPO1* mRNA in the human SSC line by TCF3-siRNA 3 and the NC-siRNA. *p < 0.05 indicated the significant differences between TCF3-siRNA 3 and NC-siRNA. **(D)** GO circle plot illustrated the top 20 enrichment terms. First circle: GO terms of the top 20 enrichments, and outside circle was the sitting scale of the number of genes. Different colors denoted the different ontologies; the second circle: the number of DEGs was enriched in this GO term and the *Q*-value. The more genes, the longer the bar, the smaller the *Q*-value, and the red the color; the third circle: the bar chart of the proportion of DEGs, and dark purple denoted the fraction of up-regulated genes. Light purple signified the proportion of down-regulated genes, and the specific value was indicated below; the fourth circle: Rich Factor value of each GO term (the number of differences in this GO term was divided by all the numbers), background grid line, each grid indicated 0.1. **(E)** KEGG circle plot for top 20 enrichment pathways. The first circle: top 20 enriched pathways, outside circle was the coordinate scale of gene number, and different colors signified different A classes; the second circle: the number of DEGs enriched in this GO term and the *Q*-value. The more genes, the longer the bar, the smaller the *Q*-value, and the red the color; the third circle: the bar chart of the proportion of DEGs, while dark purple indicated the fraction of up-regulated genes, while light purple represented the proportion of down-regulated genes, the specific value was indicated below; the fourth circle: Rich Factor value of each pathway (the number of differences in this pathway was divided by all the numbers), background grid line, each grid denoted 0.1. **(F)** Gel electrophoresis of DNA after ultrasonication. **(G,H)** qPCR evaluation of the ChIP enriched DNA samples using specified promoter or enhancer primers. Mock, negative mouse IgG control. ^∗^*p* < 0.05 indicated the significant difference from Mock.

To seek the targeting genes of the TCF3 in human SSCs, RNA sequencing was conducted to screen the alterations in transcription profiles of human SSC line by TCF3-siRNA 3 or NC-siRNA. About 19,000 genes were detected in human SSC line, and the volcano plot and heat map illustrated that 680 genes were up-regulated and 207 genes were down-regulated by TCF3-siRNA 3 (Figures 5A,B). To verify the results of RNA sequencing, a number of randomly chosen genes, including *CDON*, *SEMA3C*, *TGFB3*, *PODXL2*, *PODXL*, *TGM2*, and *RSPO1*, were determined by RT-PCR (Figure 5C), showing that and the results of these genes was consistent with RNA sequencing. GO and KEGG analyses (Figures 5D,E) demonstrated that the DEGs, including *MEGF6*, *SEMA3C*, *WNT2B*, *RPP25*, *TGFB3*, and *PODXL* (Table 1), were involved in numerous types of biological process and signaling pathways, which are critical for cell proliferation and survival.

**TABLE 1:** Top 20 down-regulated genes by TCF3-siRNA 3.

**Ensembl gene ID**	**Genes**	**NC-siRNA**	**TCF3-siRNA3**	**Fold changes**
ENSG00000099958	*DERL3*	19.63	2.49	5.94
ENSG00000162591	*MEGF6*	12.41	2.92	4.17
ENSG00000075223	*SEMA3C*	19.46	4.84	4.01
ENSG00000134245	*WNT2B*	26.27	8.75	3.17
ENSG00000075213	*SEMA3A*	62.45	21.27	3.11
ENSG00000178718	*RPP25*	22.39	7.69	3.08
ENSG00000152284	*TCF3*	111.92	38.94	3.04
ENSG00000119699	*TGFB3*	29.04	10.22	3.01
ENSG00000153993	*SEMA3D*	21.96	7.82	2.98
ENSG00000131389	*SLC6A6*	160.08	57.07	2.98
ENSG00000064309	*CDON*	28.51	10.44	2.90
ENSG00000154832	*SPP1*	65.32	24.14	2.87
ENSG00000104415	*WISP1*	99.80	37.16	2.85
ENSG00000128567	*PODXL*	162.11	62.09	2.77
ENSG00000146122	*DAAM2*	28.23	11.10	2.69
ENSG00000168743	*NPNT*	11.36	4.64	2.59
ENSG00000129128	*SPCS3*	49.48	20.23	2.58
ENSG00000149573	*MPZL2*	15.18	6.32	2.53
ENSG00000100234	*TIMP3*	239.50	100.71	2.50
ENSG00000147883	*CDKN2B*	46.95	19.82	2.49

*Gene expression values were represented by FPKM.*

TCF3 is a transcription factor of bHLH family, which can directly bind to promoter regions to regulate gene transcription. In order to identify the targeting genes which are directly regulated by TCF3, ChIP assay, and RT-PCR were performed. We predicted the binding sites of the genes, including *MEGF6*, *WNT2B*, *TGFB3*, *CCN4*, *PODXL*, *DAAM2*, and *TIMP3*, whose expression levels were down-regulated by TCF3-siRNA 3, using JasparDatabase (Fornes et al., 2020), and we designed primers of these genes (Supplementary Table 4) for the DNA sequences near the binding sites. We found that the promoter sequence of *WNT2B*, *TGFB3*, *CCN4*, *MEGF6*, and *PODXL* were significantly enriched (Figures 5F–H), and TCF3 protein can directly bind to the promoter regions of those genes to regulate their transcription.

### PODXL Is a Direct Target of TCF3 in Human SSCs

**FIGURE 6:**
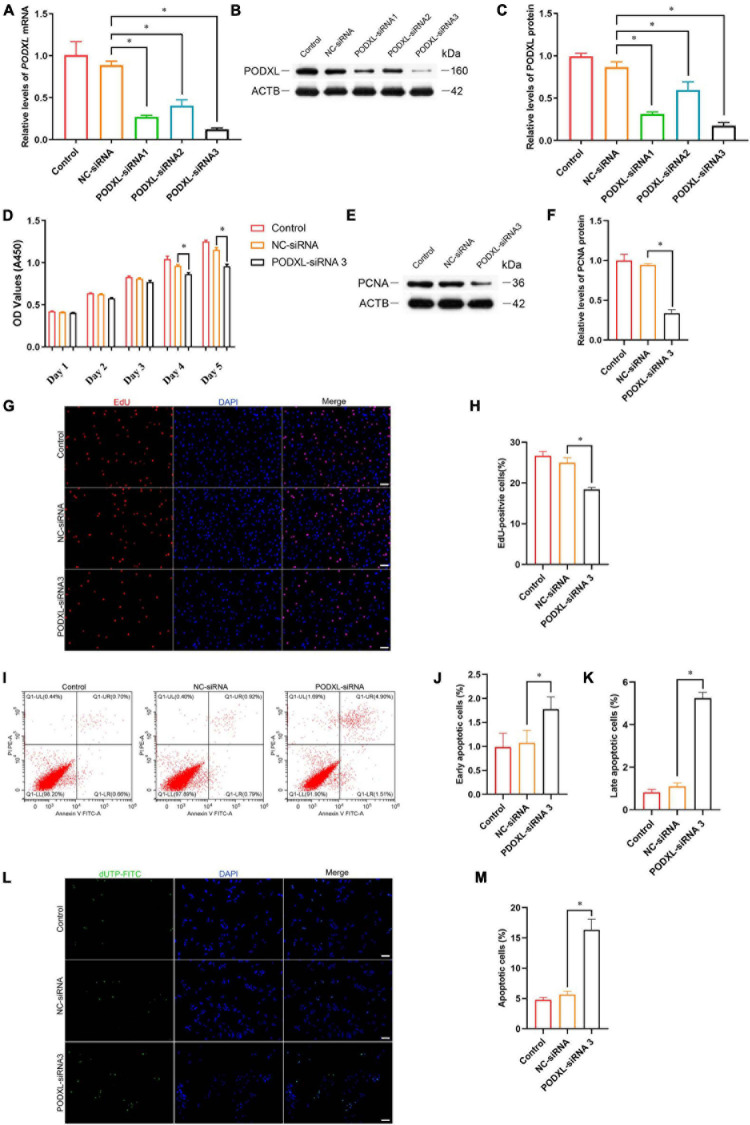
The effect of PODXL knockdown on the proliferation and apoptosis of the human SSC line. **(A)** qPCR assessment of *PODXL* mRNA in the human SSC line transfection with TCF3-siRNA 1-, 2-, and 3. *ACTB* was used for the normalization. **(B,C)** Western blotting showed PODXL proteins in the human SSC line by the PODXL-siRNA 1-, 2-, and 3 treatment. **(D)** CCK-8 assay illustrated the proliferation of in the human SSC line by NC-siRNA and PDOXL-siRNA3 treatment. **(E,F)** Western blotting revealed the PCNA protein levels in the human SSC line by the PODXL-siRNA 3 and NC-siRNA transfection. **(G,H)** EdU assays of DNA synthesis in the human SSC line with the NC-siRNA and PODXL-siRNA3 transfection. **(I–K)** Flow cytometric and APC Annexin V analysis of the proportions of early and late apoptosis in the human SSC line with the NC-siRNA and PODXL-siRNA3 transfection. **(L,M)** TUNEL assay of the fractions of TUNEL^+^ cells in the human SSC line affected by the NC-siRNA and PODXL-siRNA3. Scale bars: **(G,L)**, 50 μm. ^∗^*p* < 0.05 denoted the significant differences between PODXL-siRNA 3 and NC-siRNA.

Podocalyxin-like protein is a member of CD34 family proteins, which carries numerous post-translational epitopes responsible for various kinds of pluripotent surface biomarkers, including TRA-1-60, mAb84, TRA-1-81, GP200, and GCTM2 (Kang et al., 2016). It is also associated with more than 10 human malignancies (Kang et al., 2016; He et al., 2020). These studies suggest that PODXL may be responsible for the impaired proliferative activity of SSC by TCF3 silencing. To confirm this hypothesis, siRNA-mediated knockdown of PODXL was performed. RT-PCR and western blotting illustrated that *PODXL* mRNA and PODXL protein were reduced by all three types of PODXL-siRNA in the human SSC line, and PODXL-siRNA 3 had the most silencing effect (Figures 6A–C). CCK8 assay and EdU assay revealed that PODXL-siRNA 3 repressed human SSC proliferation, and the level of PCNA protein was reduced by PODXL-siRNA 3 (Figures 6D–H). Furthermore, flow cytometry and TUNEL assay demonstrated that the apoptosis was reduced by PODXL silencing in human SSC line (Figures 6I–M). Collectively, these data imply that PODXL knockdown represses the proliferation and DNA synthesis and promotes the apoptosis in human SSCs and that PODXL is a direct downstream target of TCF3 in human SSCs.

### TCF3 Aberrant Expression Is Associated With Spermatogenesis Failure

**FIGURE 7:**
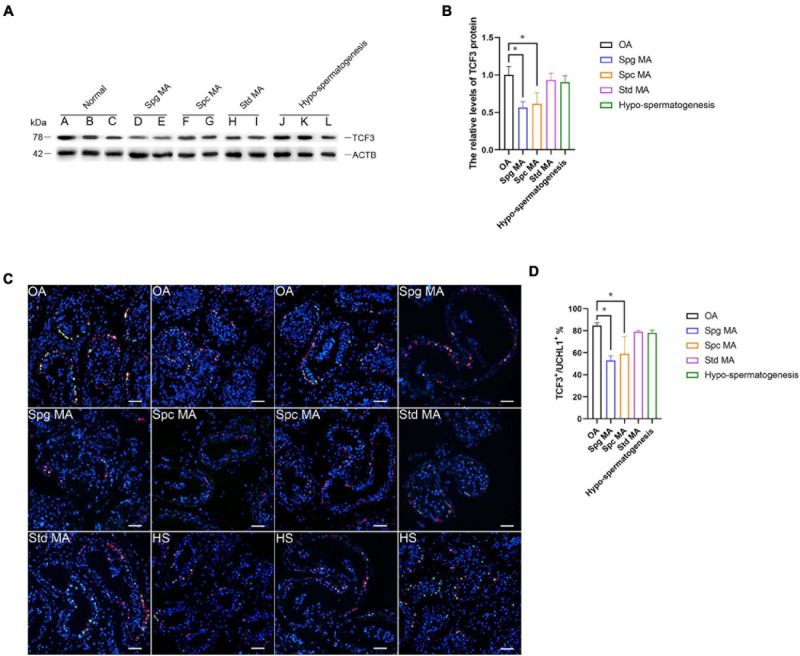
The expression of TCF3 in the testis of OA patients and NOA patients. **(A,B)** Western blotting compared the levels of TCF3 protein between OA and NOA patients. Notes in **(A)**: sample A, B, and C were individuals with OA with normal spermatogenesis; sample D and E were NOA patients with Spg MA; sample F and G were NOA patients with Spc MA; sample H and I were NOA patients with Std MA; and sample J, K, and L were NOA patients with HS. **(C,D)** The percentages of UCHL-positive SSCs with TCF3 expression between OA and various kinds of NOA patients. Scale bars: **(C)**, 100 μm. *p < 0.05 indicated the significant differences between OA patients with normal spermatogenesis and NOA patients.

NOA is a serious condition affecting male fertility. NOA with spermatogenesis failure can be classified into numerous subtypes, namely Std MA (spermatids maturation arrest), Spc MA, Spg MA (spermatogonia maturation arrest), SCOS (Sertoli cell only syndrome), and HS (hypo-spermatogenesis). To investigate the relation of TCF3 expression and spermatogenesis failure, testis tissues from 12 patients were subjected to histopathological analysis (Supplementary Figures 3A–L). We examined TCF3 expression in all testis samples, and western blotting illustrated that the level of TCF3 protein was significant lower in human testis tissues of Spg MA and Spc MA than OA patients, while the level of TCF3 was not significantly decreased in patients with Std MA and HS compared to OA patients (Figures 7A,B). Double immunohistochemistry demonstrated that the frequency of TCF3-expressing cells in UCHL1-positive SSCs was decreased in Spg MA and Spc MA (Figures 7C,D) when compared to OA patients with normal spermatogenesis. Together, these data reflect that TCF3 aberrant expression is related to maturation arrest at spermatocyte and spermatogonial stages of spermatogenesis.

## Discussion

Due to the limited availability of human testicular tissues and no effective approach of culturing and expanding human primary human SSCs, the research on human SSCs is seriously hindered. Human SSC line established by us can solve these problems, since it has an unlimited proliferation potential and the high safety, and thus this cell line is suitable for uncovering the molecular mechanism in modulating the fate decisions of human SSCs (Hou et al., 2015). It has been reported that TCF3 plays vital roles in the regulation of numerous stem cells or progenitors (Semerad et al., 2009; Pfurr et al., 2017; Yamazaki et al., 2018). Most of the phenotypes of TCF3 deficiency are linked to cell proliferation, differentiation, or death. Nevertheless, the impact of TCF3 on stem cell self-renewal seems to depend on the cell types. In human testes, we revealed that TCF3 was primarily or specifically expressed in self-renewing SSCs (GFRA1^+^/KIT^–^), which was consistent with the finding, by the single cell sequencing, that TCF3 is expressed in cells at States 0 and 1 with a high similarity to infant SSCs or SSEA4-positive adult SSCs, respectively (Guo et al., 2018). We also found that *TCF3* mRNA was up-regulated by EGF, EGF promotes cell proliferation *via* RAS or PI3K pathway (Margolis and Skolnik, 1994; Sabbah et al., 2020), which are key pathways regulating the self-renewal of SSCs in rodents (Kanatsu-Shinohara and Shinohara, 2013), but the precise functions and mechanisms in human spermatogonia are yet to be defined. The leydig cells are the principal source of EGF in the testis, germ cells also produce EGF at the onset of sexual maturation and spermatogenesis (Wald, 2005). Our work will help to explain how the microenvironment support SSC self-renewal and the importance of the cell crosstalk.

Significantly, we demonstrated that TCF3 stimulated the proliferation and DNA synthesis of human SSCs, whereas it inhibited the apoptosis of human SSCs, which would be helpful to expand human SSCs *in vitro* to obtain sufficient cells for basic research and translational application of human SSCs. By RNA sequencing and ChIP assays, we revealed that TCF3 protein regulated the transcription of several genes, including *WNT2B*, *TGFB3*, *CCN4*, *MEGF6*, and *PODXL*. Interestingly, CDH1, which is a target of TCF3 in ESCs (Yamazaki et al., 2018) and it can regulate the self-renewal through cell adhesion of mouse SSCs (Tokuda et al., 2007), was not significantly decreased by TCF3 siRNA in human SSC line, which indicates that CDH1 is not a target for TCF3 in human SSCs. Other genes involved in cell adhesion, including *CDH3*, *PCDH7*, *TGM2*, and *PODXL*, were significantly reduced by TCF3 siRNA in human SSC line. Those data suggest that TCF3 may influence the adhesion of human SSCs *via* different downstream targets compared to ESCs. It has been shown that PODXL is a pluripotent surface marker and enriched in many cancer cells (He et al., 2020), PODXL positive hematopoietic stem cells can reconstitute myeloid and lymphoid lineages in recipients with lethal radiation (Doyonnas et al., 2005). PODXL promotes cancer development and aggressiveness by activating MAPK and PIK3 pathways. Those evidences indicates that PODXL may be related to stemness and cell proliferation. E- and L-selectin were reported as functional ligands in cancer cells, but we are not sure whether L- or E-selectin can promote SSC proliferation through PODXL. We observed that PODXL silencing compromised the stem cell activity of human SSCs, the roles of other candidate target genes of TCF3 need to be further explored. TGFB3 is a TGF-β (transforming growth factor-beta) family member. TGF-β members are multifunctional cytokines, which participate in the modulation of numerous biological processes, namely cell proliferation, survival, and differentiation (Young et al., 2015). TGFB3 is believed to modulate BTB (blood-testis barrier) dynamics by promoting the endocytosis of integral membrane proteins and their intracellular degradation (Wong et al., 2010), which may be associated with male infertility (Droździk et al., 2015). Given that TGFB3 stimulates adipocyte progenitor proliferation (Petrus et al., 2018), it is possible that TGFB3 may also affect spermatogenesis by regulating the proliferation of SSCs. Interestingly, it has been shown that the suppression of the TGFB1 receptor enhances spermatogonial proliferation and spermatogenesis (Moraveji et al., 2019). WNT2B is a ligand of the frizzled family and functions in the canonical Wnt/beta-catenin signaling pathway (Goss et al., 2009), the effect of WNT2B deletion was not as obvious as that of PODXL (data not shown). Although Wnt/β-catenin signaling pathway has been proved to promote the differentiation of in mouse SSCs (Tokue et al., 2017), as a secretory protein, WNT2B may help for our understanding of crosstalk between spermatogonia. SLC6A6 is a transporter of taurine and beta-alanine, which is critical for material transport and energy metabolism (Han et al., 2006), although we did not find evidence that TCF3 could bind to the promoter region of SLC6A6, we are interested in the functions of SLC6A6 in spermatogenesis, it may provide cues to understand the energy metabolism of SSCs.

The TCF3 protein was initially discovered in B-cells as immunoglobulin enhancer binding proteins (Rutherford and LeBrun, 1998); it has a core role in the transcriptional modulator networks that the enhance commitment and differentiation of the B and T cell lineages, and their role in these lineages is regulated by inhibitor of DNA binding (ID) proteins. TCF3 protein can dimerize with any of the four ID proteins (ID1-ID4) (Kee, 2009), and the stimulation of ID3 expression in T cells antagonizes the DNA binding activity of TCF3. Moreover, it is suggested that ID4 is a distinguishing marker of SSCs in the mammalian germline and it modulates the self-renewal of SSCs (Oatley et al., 2011; Chan et al., 2014). Given that TCF3 proteins can dimerize with ID4, and both TCF3 and ID4 are expressed selectively by SSCs, it is possible that TCF3 and ID4 orchestrate the SSCs fate determination and maintain the stem cell pool. Nevertheless, the interactions between TCF3 and ID proteins in SSCs remain to be explored further, while our results provide clues for understanding the functions of ID4 and TCF3.

We have also illustrated that the expression level of TCF3 was reduced in NOA patients, especially in patients with maturation arrest at spermatogonial and spermatocyte stages. Consistent with our observations, a transcription profiling data of human spermatogenic failure also reveals that *TCF3* is down-regulated in spermatogonial maturation arrest and SCOS patients (Spiess et al., 2007); but there is no significant difference in spermatocyte arrest patients (Spiess et al., 2007). We found that there might be the correlation between TCF3 low level and spermatogenic failure. Thus, it would be interesting to establish animal models to further determine whether the deficiency of TCF3 will cause spermatogenesis failure and male infertility.

## Conclusion

In summary, we have demonstrated, for the first time, that TCF3 is primarily expressed in human SSCs and that it mediates the proliferation, DNA synthesis, and apoptosis of human SSCs. We also unveil an association between TCF3 expression and spermatogenesis failure. Furthermore, we reveal that TCF3 is regulated by EGF and PODXL is a target of TCF3 in human SSCs. Therefore, this study provides novel genes and regulators in modulating the fate determinations of human SSCs. This study is of particular significance since it could offer new targets for gene therapy of male infertility.

## Data Availability Statement

The original contributions presented in the study are included in the article/Supplementary Material, further inquiries can be directed to the corresponding authors.

## Ethics Statement

This study was approved by the Ethics Committee of the Reproductive & Genetic Hospital of CITIC-Xiangya, Basic Medical Science School, Central South University (LL-SC-2017-015). The patients/participants provided their written informed consent to participate in this study.

## Author Contributions

LF and ZuH designed the study and supervised the laboratory experiments. DZ conducted the experiments and drafted the manuscript. JF, ZL, RT, XW, HB, FZ, XZ, ZeH, LX, and KT assisted with the experiments and sample collection. HN, HaZ, HuZ, and WZ performed the analysis with constructive discussion and contributed analysis tools. All authors contributed to the article and approved the submitted version.

## Conflict of Interest

The authors declare that the research was conducted in the absence of any commercial or financial relationships that could be construed as a potential conflict of interest.

## Publisher’s Note

All claims expressed in this article are solely those of the authors and do not necessarily represent those of their affiliated organizations, or those of the publisher, the editors and the reviewers. Any product that may be evaluated in this article, or claim that may be made by its manufacturer, is not guaranteed or endorsed by the publisher.
