# Supplementary material for: TCF3 Regulates the Proliferation and Apoptosis of Human Spermatogonial Stem Cells by Targeting PODXL
Source: Front Cell Dev Biol. 2021 Aug 6;9:695545. doi: 10.3389/fcell.2021.695545 (PMC8377737; doi:10.3389/fcell.2021.695545)
Supplement: Supplementary file 1 [file Presentation_1.pdf]

Supplemental Tables

Table S1. The primers used for real-time PCR and RT-PCR

Genes	Primer sequences (5'-3')	Product sizes (bp)
TCF3	F: GCACTGGCCTCGATCTACTC	172
	R: TCTTACTCAGGCCGTGGAGA	
SV40	F: GAACAGCCCAGCCACTATAA	248
	R: ACTCCAGCCATCCATTCTTC	
THY1	F: ATCGCTCTCCTGCTAACAGTC	135
	R: CTCGTACTGGATGGGTGAACT	
GFRα1	F: CGGGTGGTCCCATTCATATC	411
	R: TGGCTGGCAGTTGGTAAA	
RET	F: CTCGTTCATCGGGACTTG	126
	R: ACCCTGGCTCCTCTTCAC	
PLZF	F: CGGTTCCTGGATAGTTTGC	317
	R: GGGTGGTCGCCTGTATGT	
GPR125	F: GCGTCATTACGGTCTTTGGAA	199
	R: ACGGCAATTCAAGCGGAGG	
UCHL1	F: AGCTGAAGGGACAAGAAGTTAG	265
	R: TTGTCATCTACCCGACATTGG	
GATA4	F: GCCTCCTCTGCCTGGTAAT	120
	R: CAGTCCCATCAGCGTGTAAA	
SOX9	F: AGGTGCTCAAAGGCTACGACTG	322
	R: TGCCCGTTCTTCACCGACT	
VASA	F: GCAGAAGGAGGAGAAAGTAGTG	289
	R: CTCGTCCTGCAAGTATGATAGG	
ACTB	F：CGCACCACTGGCATTGTCAT	206
	R: TTCTCCTTGATGTCACGCAC	
PODXL	F:TCCCAGAATGCAACCCAGAC	179
	R:GGTGAGTCACTGGATACACCAA	
CDON	F:CAGAAACTTGGTGGACCTGTAG	75
	R:GTTATGCAGCCATGAGATACGA	
SEMA3C	F:TTTGCGTGTTGGTTGGAGTAT	238
	R:TCCTGTAGTCTAAAGGATGGTGG	
TGFB3	F:AACGGTGATGACCCACGTC	119
	R:CCGACTCGGTGTTTTCCTGG	
PODXL2	F:CTCCCTGCTAGACCTCCTG	144
	R:TGCAGAATCCGAGACTCTTCAT	
TGM2	F:GAGGAGCTGGTCTTAGAGAGG	184
	R:CGGTCACGACACTGAAGGTG	
RSPO1	F: TGGAGAGGAACGACATCCG	156
	R:CCTTACACTTGGTGCAGAAGTTA	

F: forward primer; R: reverse primer

Table S2. Antibodies applied in Western blots, immunofluorescence and immunoprecipitation

Antibodies	Source	Dilution
Western blots		
TCF3	Sigma cat#hpa062476	1:400
GPR125	Abcam cat#ab51705	1:500
UCHL1	Abcam cat#ab8189	1:400
THY1	Abcam cat#ab133350	1:500
PLZF	R&D cat#AF2944	1:500
GFRα1	R&Dcat#AF560	1:200
PCNA	Abcam cat#ab29	1:500
PODXL	Abcam cat#ab150358	1:400
Immunofluorescence		
TCF3	Sigma cat#hpa062476	1:100
GPR125	Abcam cat#ab51705	1:100
UCHL1	Abcam cat#ab8189	1:50
THY1	Abcam cat#ab133350	1:100
PLZF	R&D cat#AF2944	1:50
GFRα1	R&Dcat#AF560	1:25
PCNA	Abcam cat#ab29	1:50
KIT	R&D cat#AF332	1:25
DDX4	Abcam cat#ab27591	1:100
Secondary Antibody		
Donkey anti-Rabbit IgG,Alexa Fluor 488	Thermo Fisher Scientific cat#A21206	1:1000
Donkey anti-Mouse IgG,Alexa Fluor 594	Thermo Fisher Scientific cat#A21203	1:1000
Donkey anti-Goat IgG,Alexa Fluor 594	Thermo Fisher Scientific cat#A11058	1:1000
Immunoprecipitation		
TCF3	Santacruz cat#133075	1:10

Table S3. The siRNA sequences targeting human TCF3 and PODXL mRNA

siRNA	Targeting sequence
TCF3-siRNA1	CCGGATCACTCAAGCAATA
TCF3-siRNA2	GAACCTGAATCCCAAAGCA
TCF3-siRNA3	AGCCTCTCTTCATCCACAT
PODXL-siRNA1	ACATGACCATCTTATGAAA
PODXL-siRNA2	GGACTCATCTAACAAAACA
PODXL-siRNA3	AGACCGTGGTCGTCAAAGA

Table S4. The promoter sequence primers used for ChIP-qPCR

Genes	Forward primers	Reverse primers
TGFB3-1	CATGGACAAGAATCCCAGCG	GAGAGCTTCAGGACTTCCAGG
TGFB3-2	GGCGTGCGAGAGAAGGAATA	ACTTTGTTCACGCTGCCTCT
TGFB3-3	GAGGCAGCGTGAACAAAGTC	TGAGTAGGTGGGGAGAAGCA
WNT2B-1	ACGAAGGAGGGAAAAAGCTCC	GGGTGCTTTTCTTTACGGACC
WNT2B-2	CACCCATAGAAGTGGGGCTG	GACGCCCTAGGTGTAGCAG
CCN4-1	TAAGAACTGTCAGAGCTGGGG	TGACGTCAGGGTGAAGGACAA
CCN4-2	CTGGGAGCCCTCTCAAAGC	ATGGGAGGGTGCAGGATGTC
PODXL-1	GCAGGTGGGAGATGAGCTTC	CAGCCTTGCGTGGTAACTGA
PODXL-2	GCTCCGGATTTGCTCGTAGT	CCCGAGTGCTTCTAAGTGGG
DAAM2	CATTTGCCCGTAGCCCAGAG	CGAGGCGTACAATGAGGGTA
TIMP3	GCAAACAGCAGATGGCTTCC	CCTTGACTGTGCTTGGTGGA
MEGF6	CGTGTTGGCTCCAAGAACAC	GACCTAGGCACAAACGCAGA

Supplemental Figures

Supplemental Figure 1

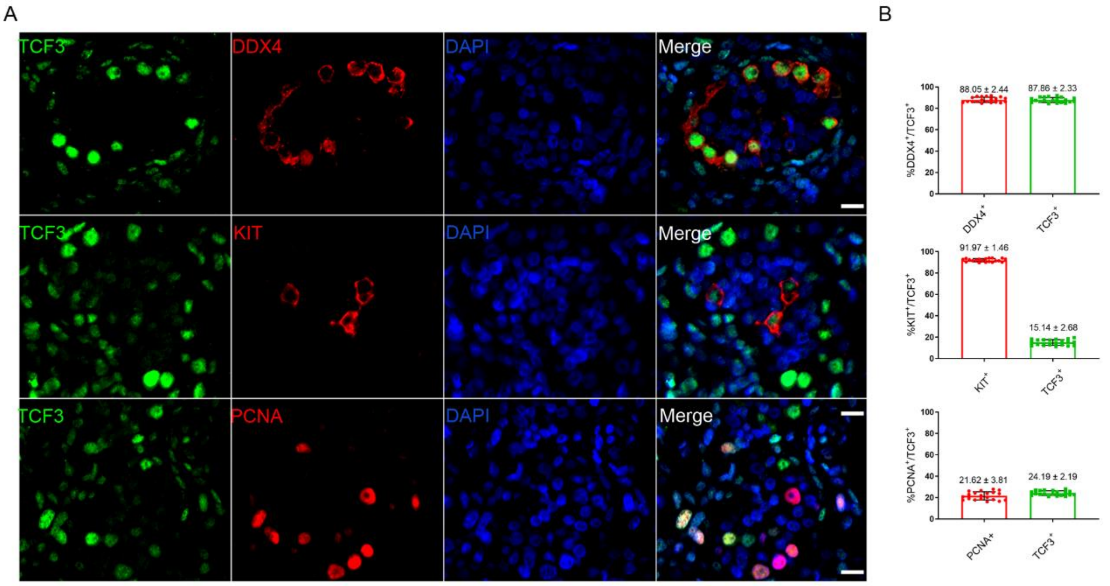

Figure S1. The expression of TCF3 in human fatal testis. (A) Double immunostaining revealed the co-expression TCF3 with DDX4, KIT and PCNA in fetal testis. At least 20 tubules were counted. (B) Percentages of $TCF3^+$ cells with DDX4, KIT and PCNA expression. Scale bars: A, 20 μm.

Supplemental Figure 2

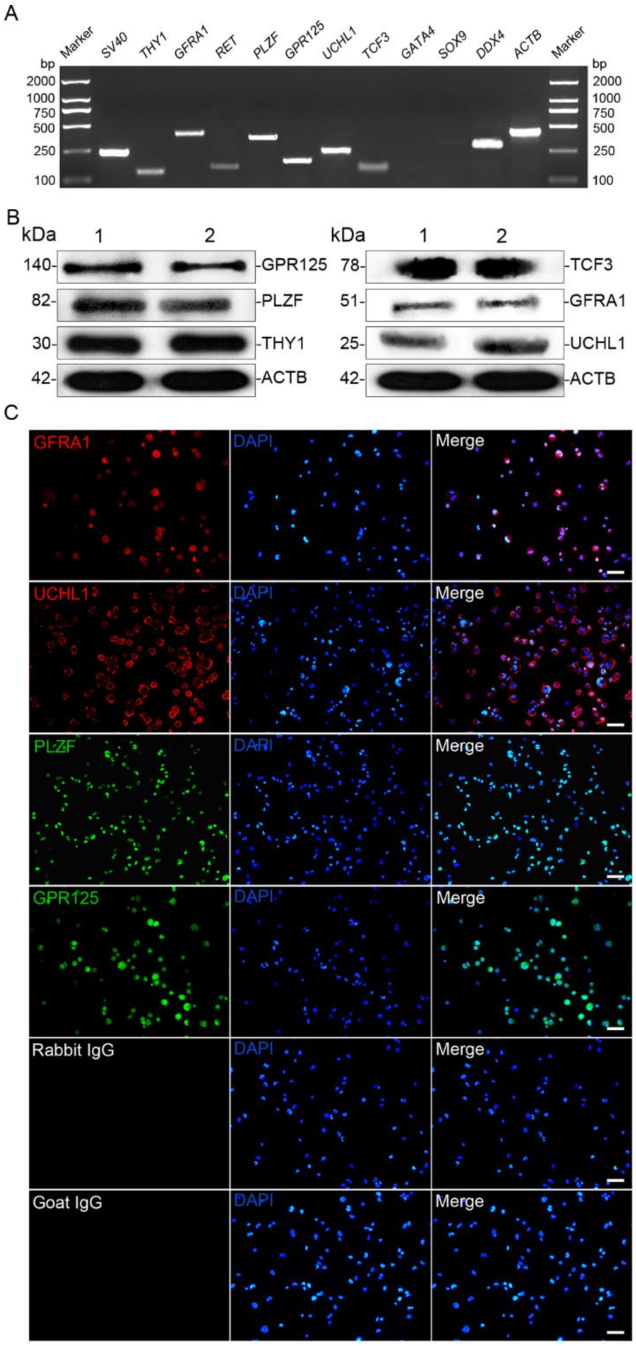

Figure S2. Identification of the Human SSC line. (A) RT-PCR revealed gene expression of *THY1*, *GFRA1*, *RET*, *PLZF*, *GPR125*, *UCHL1*, *TCF3*, *GATA4*, *SOX9* and *DDX4* in the human SSC line. Sertoli cell marker genes *GATA4* and *SOX9* were not detected, and *ACTB* served as the loading control of total RNA. **(B)**Western blots showed the protein expression of GPR125, TCF3, PLZF, GFRA1, THY1 and UCHL1

in human SSC line. ACTB was used as the control of loading proteins. 1 and 2 represented two independent human SSC line samples. **(C)** Immunocytochemistry demonstrated the presence of GFRA1, UCHL1, PLZF and GPR125 in human SSC line. Normal rabbit IgG and goat IgG served as negative controls. Scale bars: C, 50 μm.

Supplemental Figure 3

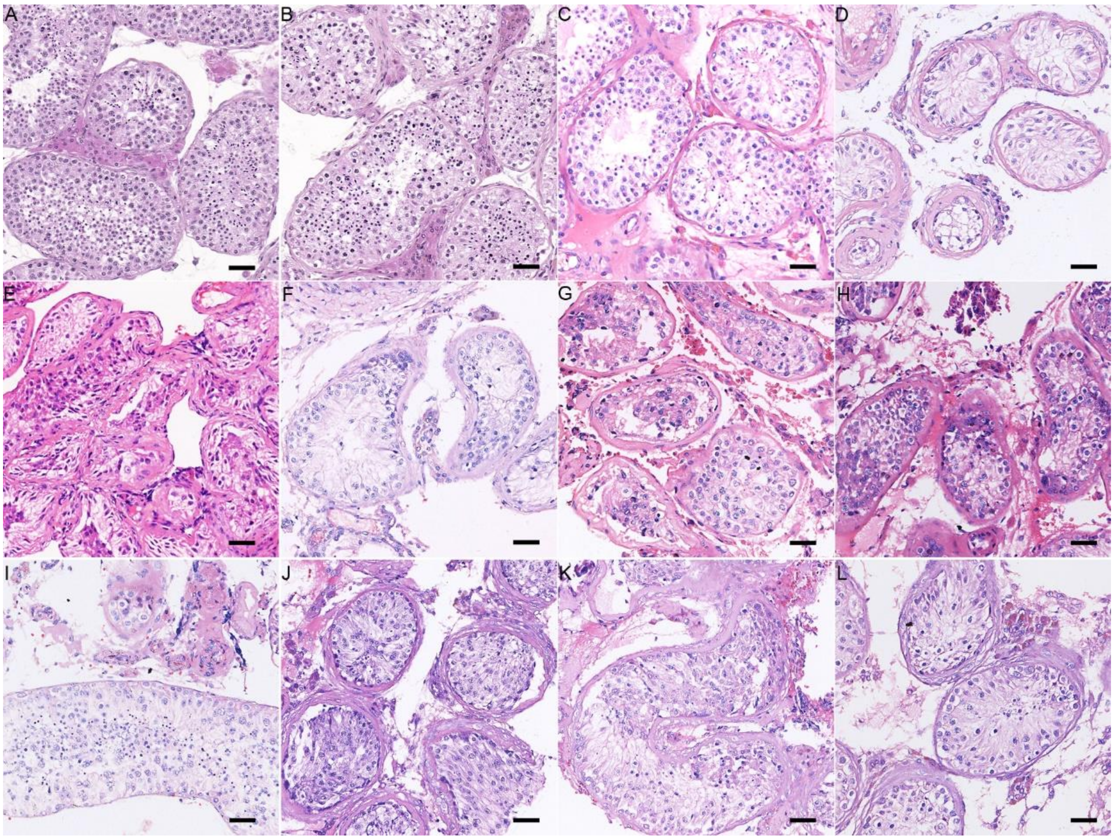

Figure S3. H&E staining for testes of OA and NOA patients with germ cell maturation arrest. (A-C) Testis samples from OA patients with normal spermatogenesis, and numerous spermatozoa were observed in seminiferous tubules. (D and E) Testis samples from NOA patients with spermatogonial maturation arrest, and only few spermatogonia and Sertoli cells were seen in the seminiferous tubules. (F and G) Testis samples from NOA patients with spermatocyte maturation arrest, and there were a number of spermatocytes and no spermatid. (H and I) Testis samples from NOA patients with spermatid maturation arrest. While there were some spermatids in seminiferous tubules, there was no elongated sperm. (J-L) Testis

samples from NOA patients with hypo-spermatogenesis, and a very small amount of sperm was observed in some tubules. Scale bars: A-L, 100 μm.
